# Food Pattern, Food Selectivity and Sensory Profile in Autism Spectrum Disorder: An Exploratory Analysis in Chilean Children

**DOI:** 10.3390/children12111560

**Published:** 2025-11-17

**Authors:** Fernanda Mora, María José Manzur, David Morales-Zepeda, Oscar Flores, Constanza Schwencke, Marcell Leonario-Rodriguez

**Affiliations:** 1 Escuela de Nutrición y Dietética, Facultad de Medicina y Ciencias de la Salud, Universidad Mayor, Santiago 8580745, Chile; fernanda.mora@mayor.cl (F.M.); maria.manzur@mayor.cl (M.J.M.); 2 Centro de Investigación en Sociedad y Salud, Universidad Mayor, Santiago 8580745, Chile; david.moralesz@mayor.cl; 3 Academia Científica de Estudiantes de Nutrición y Dietética (ACENUM), Facultad de Medicina y Ciencias de la Salud, Universidad Mayor, Santiago 8580745, Chile; oscar.floresr@mayor.cl (O.F.); constanza.schwencke@mayor.cl (C.S.)

**Keywords:** autism, autism spectrum disorder, food selectivity, hypersensory profile

## Abstract

**Background/Objectives:** Introduction: Food selectivity is highly prevalent in children with autism spectrum disorder (ASD) and is associated with sensory hypersensitivity, particularly in oral, olfactory, and tactile domains. Although international evidence exists, little is known about this phenomenon in Latin American populations. This study aimed to explore the relationship between sensory hypersensitivity and food selectivity in Chilean children with and without ASD. **Methods:** A cross-sectional study was conducted with 57 children aged 6–12 years, including 32 with ASD and 25 neurotypical controls. Sensory processing was assessed using the Dunn Sensory Profile 2, while food selectivity was evaluated with the Brief Autism Mealtime Behaviour Inventory (BAMBI) and a Food Frequency Questionnaire (FFQ). Statistical analyses included intergroup comparisons and correlation tests. **Results:** Children with ASD obtained significantly higher scores across all domains of sensory hypersensitivity (*p* < 0.001). Selective eating behaviours were more frequent, with texture (78%) and colour (53%) being the most common, and were strongly associated with ritualistic eating (OR 29.39; 95% CI 5.47–136.2; *p* < 0.0001). BAMBI scores were correlated with oral (*p* = 0.002), socio-emotional (*p* = 0.003), and somatic hypersensitivity (*p* = 0.025). Additionally, children with ASD reported lower intake of vegetables, dairy products, animal proteins, and legumes compared with controls. **Conclusions:** Food selectivity in Chilean children with ASD is closely related to sensory hypersensitivity, particularly in oral, socio-emotional, and somatic domains. These findings underscore the need for culturally adapted, sensory-based interventions to broaden dietary variety and reduce mealtime difficulties in this population.

## 1. Introduction

Autism Spectrum Disorder (ASD) is a neurodevelopmental condition that emerges in early stages of life and is characterised by persistent difficulties in communication and social interaction, as well as by restrictive and repetitive patterns of behaviour, interests, or activities, which significantly impact quality of life [1]. Its etiology arises from a complex interaction between genetic and environmental factors, positioning it as one of the most prevalent neurodevelopmental disorders worldwide [2]. At the global level, the prevalence is estimated at 0.72%, with higher rates reported in North America (1.01%) and among children aged 6 to 12 years (0.82%), showing a sustained increase since the 1990s [3]. In Chile, a prevalence of 1.06% was reported in the general population in 2021 [4].

In terms of development, children with ASD exhibit generalised impairments across cognitive, motor, adaptive, socio-emotional, and communicative domains, frequently accompanied by disruptive behaviours [5]. These difficulties, particularly in the social domain, are associated with an increased risk of developing mood disorders such as anxiety and depression, as well as greater functional dependence in adulthood, limiting personal autonomy and social integration [6].

Beyond socio-emotional functioning, these challenges may also manifest in the dietary domain. Children with ASD often display problematic mealtime behaviours, such as refusing certain foods, showing low acceptance of fruits and vegetables, leaving the table, or resisting trying new foods, which creates a tense environment and increases family stress [7]. Food selectivity is highly prevalent among children with ASD, affecting between 46% and 89% [8]. This condition is characterised by rejection of foods with mixed, sticky, or blended textures, along with a clear preference for foods with a uniform structure, a crunchy consistency, and neutral colours. This may be attributed to heightened sensory hypersensitivity in the domains of taste, smell, and touch, in which both sensory characteristics and stereotyped behavioural patterns significantly contribute to this selectivity [9]. Oral hypersensitivity is a key risk factor for food selectivity in children with ASD, as it is associated with limited dietary variety, thereby reinforcing the link between sensory symptoms and restrictive eating patterns. Olfactory hypersensitivity may increase avoidance of foods with intense or unfamiliar aromas. In contrast, somatic/tactile hypersensitivity (e.g., aversion to mouthfeel) and visual sensitivity (e.g., strong responses to colour uniformity) can further reinforce rigid preferences (e.g., crunchy, single-texture, neutral-colored foods) and feeding rituals. These domain-specific sensitivities plausibly converge on food selectivity via heightened sensory defensiveness and behavioural rigidity, thereby constraining exposure to diverse foods and limiting dietary quality [10,11]. From a theoretical perspective, sensory integration models propose that atypical sensory processing in ASD can disrupt the regulation of feeding behaviours through heightened sensory defensiveness and reduced habituation to novel stimuli. These mechanisms foster rigid food preferences and avoidance behaviours, reinforcing selective eating patterns and narrowing dietary diversity. Empirical evidence supports these associations: children with ASD who display greater sensory hypersensitivity tend to have narrower dietary repertoires and lower fruit and vegetable intake, highlighting a clear link between sensory defensiveness and behavioural rigidity [12,13].

Despite the growing international literature on ASD and sensory processing, studies in South American populations remain scarce. This gap is particularly relevant given the region’s sociocultural and dietary specificities, which may shape both sensory responsiveness and feeding patterns. In Chile, typical meal structures, texture preferences, and the availability of staple foods may influence children’s sensory experiences. At the same time, disparities in access to occupational therapy and caregiver support could further modulate these relationships. Addressing this gap is crucial to diversifying the global evidence base and highlighting the unique characteristics of underrepresented populations [14,15].

A deeper understanding of these mechanisms could provide more effective tools for healthcare professionals who must address food selectivity in this population, particularly in underrepresented contexts such as South American children with ASD. In this regard, the objective of the present study was to evaluate the relationship between different sensory hypersensitivity domains and food selectivity patterns in Chilean children with and without an ASD diagnosis.

This study is grounded in the preliminary assumption that the Chilean sociocultural and dietary context modulates the expression of food selectivity. This justifies the need for the present study, given the potential lack of applicability of predominantly Anglo-Saxon evidence. In doing so, we aim to establish a crucial baseline for sensory profiles in ASD within South America, thereby contributing to a more diverse global understanding of the disorder. Furthermore, this work aims to facilitate replication in neighbouring countries and guide the development of future culturally adapted clinical interventions.

## 2. Materials and Methods

### 2.1. Study Design

A cross-sectional observational study was conducted among school-aged children and preadolescents, of whom 32 had a diagnosis of ASD and 25 presented neurotypical development, to identify specific patterns in the population of interest. Recruitment and data collection were carried out through contact with parents and caregivers associated with four private foundations and institutions in the Metropolitan Region and the Region of La Araucanía, Chile, during 2023 and 2024, which are focused on providing therapeutic support to children with ASD. The study design was based on previously published work in the field [9].

### 2.2. Participants

The pediatric population with ASD was required to meet the following eligibility criteria: a confirmed diagnosis by a pediatric neurologist based on DSM-5 criteria, male or female sex, age between 6 and 12 years, and enrollment in an institution with some form of therapy. The research team did not administer a standardized diagnostic assessment to reconfirm the diagnosis, as the participating institutions conducted this procedure upon each child’s admission, based on a neurologist’s diagnosis according to DSM-5 criteria. For the neurotypical children serving as the control group, both sexes aged 6 to 12 years were included. Regarding exclusion criteria, participants with any chronic diagnosis of cardiometabolic diseases, gastrointestinal disorders, or nutritional treatment, or with acute conditions that could alter usual food intake at the time of assessment, were excluded.

The required sample size was estimated based on reference values from a physiological parameter widely documented at both national and international levels, given the absence of prior data for the main study variables (food selectivity and sensory hypersensitivity) in ASD populations [16]. This parameter was used solely as a methodological reference to obtain a conservative and standardized estimate of the minimum number of participants required, as it provides reliable normative data and consistent population variability. Its use does not imply any conceptual or functional relationship with the studied phenomena but instead responds to methodological validity, feasibility, and statistical robustness criteria [17].

The calculation was performed using G*Power 3.1 software (University of Düsseldorf, Düsseldorf, Germany). The significance level was set at 5%, the statistical power at 80%, and the effect size at 0.8, according to Cohen’s d, resulting in an estimated sample size of 25 participants per group. For categorical contrasts (e.g., the prevalence of the “More than others” category in the Sensory Profile 2 or high BAMBI scores), detecting moderate-to-large differences in proportions (e.g., 40% vs. 70%) would require approximately *n* ≈ 42 per group. Given the exploratory nature of the study and feasibility constraints, the achieved sample (*n* = 57) provides adequate statistical power to detect moderate effects in correlational analyses and large effects in group comparisons.

### 2.3. Procedures

As part of the recruitment process, an email was sent to the directors of foundations for children with ASD, inviting them to participate in the study. Upon authorisation, the directors provided a database of the children’s caregivers’ telephone contacts, who had previously been informed about the initiative. Based on this, field researchers contacted the caregivers by phone to obtain authorisation. When the response was positive, the participant was enrolled in the study. If no response was received within one week, a follow-up message was sent. In the absence of a reply after this second attempt, no further contact was made.

It is important to note that each researcher held a Bachelor’s degree in Nutrition and Dietetics at the time of participation in the study and had prior training in data collection. To ensure that the field researchers were aligned with the pre-established data collection guidelines, a pilot simulation session was conducted. During this session, the principal investigator joined the video conference to observe the process and provide feedback on areas requiring improvement. For children without an ASD diagnosis, the same procedure was replicated in schools within the same geographic area.

Following a positive response from caregivers, a 60-min videoconference session was scheduled within a short timeframe. During this session, the informed consent form was presented and read together; participation was accepted via online signature; and the study instruments were subsequently administered. The tools were digitised in a format accessible only to the researchers, and this was completed in real time during the online session. Results were tabulated immediately by the platform (Google Forms^®^) and could be downloaded only by the principal investigator (M.L.-R.), who curated, processed, and analysed the data.

All parents and caregivers who participated in the study signed an informed consent form describing the study’s objectives and procedures before enrollment. Furthermore, the entire project was conducted in accordance with the principles of the Declaration of Helsinki and was approved by the Scientific Ethics Committee of Universidad Mayor, Chile (Folio 0326).

### 2.4. Measure Variables

Using a self-developed instrument, information was collected on general background (age, sex, type of institution, type of therapy), as well as on sensory processing and food selectivity. The description of the tools used for the main variables is provided below.

#### 2.4.1. Sensory Processing and Hypersensitivity

To evaluate this variable, the Sensory Profile 2 developed by Winnie Dunn was used [14]. This tool assesses children’s sensory processing patterns by administering 86 items to parents, caregivers, or teachers. The responses are categorised into nine processing domains (Auditory, Visual, Tactile, Movement, Body, Oral, Behavioural, Socio-emotional, and Attentional). Answers range from Never to Always and are scored on a 5-point scale, with each domain yielding a score that indicates whether the child’s processing is classified from “Much less than others” to “Much more than others.” For the present study, the classification “More than others” was used as the cutoff point, with specific threshold scores established for each domain (Supplementary Materials Table S1). It is important to note that the instrument has been validated for Spanish-speaking populations and for the age group considered in the present study. It is also widely used in clinical settings in Chile and in research contexts [18,19].

#### 2.4.2. Food Selectivity and Food Pattern

To determine the degree of food selectivity, the Brief Autism Mealtime Behaviour Inventory (BAMBI) and the Food Frequency Questionnaire (FFQ) were administered. The former is an 18-item tool answered on a Likert scale ranging from *Never* to *Always*. Items are scored from 1 to 5, except for three items that are reverse-scored. Higher scores indicate more problematic mealtime behaviours [20]. The FFQ is a widely used tool for collecting dietary information and consists of a questionnaire that records the portions of foods consumed regularly. Responses are provided in terms of daily, weekly, biweekly, or monthly intake and are structured by food groups. In this case, the questionnaire included cereals, fruits, vegetables, dairy products, meats, legumes, oils and fats, sugars, snacks, and beverages [21]. Concerning the FFQ, it has been validated for Spanish-speaking and Chilean populations and is widely used in clinical and community practice in the country. In contrast, the BAMBI does not have a national validation, and there are no comparable instruments available for Chilean ASD populations, as this group remains underrepresented. Nevertheless, several studies have employed this tool in research contexts [22,23].

### 2.5. Data Analysis

For data management, spreadsheets were generated in Microsoft Excel^®^, from which data were cleaned and curated before subsequent processing in GraphPad Prism^®^ v.9.5.1 (San Diego, CA, USA). Proportions for each variable were calculated to characterise the sample, along with the mean, standard deviation, and 95% confidence intervals for the continuous quantitative data obtained for each group. To determine significant differences in scores by ASD diagnosis, data normality was first assessed, and the Mann–Whitney U test was then applied. In addition, the correlation between food selectivity and sensory hypersensitivity by domain was evaluated using Spearman’s test.

## 3. Results

Information was successfully collected from 57 children (Table 1), the majority of whom were male (~58%), belonged to the 6–9 years age group (~77%), and had a diagnosis of ASD (~57%). For attendance at educational institutions, the vast majority (84%) were enrolled in traditional schools. Sixty per cent reported attending therapy, with the most common being occupational therapy, psychology, and speech therapy (33–39%), and, to a lesser extent, medical or physical treatment (<9%). None of the participants included in the study attended a nutritionist. As for whether caregivers could identify food aversions based on their characteristics, approximately 57% reported aversion to textures, 44% to the smells of preparations, and 35% to specific colours. In addition, regarding practices repeated during mealtime, 44% reported their presence.

For the association between ASD diagnosis and selectivity based on food characteristics and practised rituals (Table 2), significant results were observed. First, the practice of mealtime rituals showed the strongest association (OR 29.39; 95% CI 5.47 to 136.2; *p* < 0.0001), followed by aversion or preference based on food texture, with a similar magnitude of statistical significance (OR 9.18; 95% CI 2.78 to 27.5; *p* = 0.0002). Colour was also identified as a factor influencing food preferences in children with ASD compared to those without the condition, although to a lesser extent than the previously described variables (OR 8.31; 95% CI 2.07 to 29.4; *p* = 0.0018). About the smell of preparations, an association with ASD diagnosis was found; however, it did not reach statistical significance (*p* = 0.1783).

When comparing dietary patterns between neurodivergent and neurotypical children across food groups (Table 3), differences emerge depending on the nature of the foods. For example, cereal and fruit intake was quite similar between both groups of children (*p* > 0.05). The situation differed, however, for specific foods such as vegetables (lettuce *p* = 0.002; tomato *p* = 0.016), dairy products (soft cheese *p* = 0.037), animal proteins (meat *p* = 0.021; pork *p* = 0.003), legumes (green peas *p* = 0.005), fats and oils (margarine *p* = 0.048; avocado *p* = 0.007), sugars and snacks (sucrose *p* = 0.015), and beverages (water *p* = 0.048; carbonated drinks *p* = 0.005), where intake was consistently higher among neurotypical children compared to neurodivergent children.

**Figure 1:**
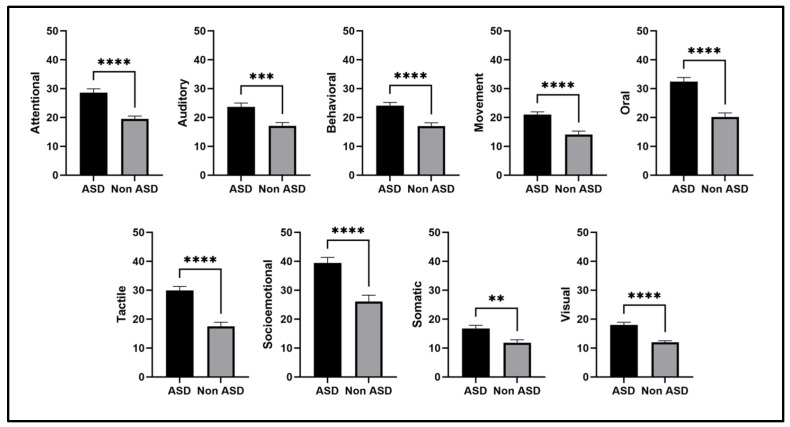
Sensory Hypersensitivity Profiles in ASD and Non-ASD Populations. Data are presented as mean ± SEM. Statistical comparisons were performed using the Mann–Whitney test (** *p* < 0.01, *** *p* < 0.001, **** *p* < 0.0001).

When analysing the different domains of sensory hypersensitivity according to ASD diagnosis (Figure 1), neurodivergent children showed higher sensitivity values across the nine domains measured by the scale than their neurotypical peers. More pronounced differences were reported in the attentional, behavioural, movement, oral, tactile, socio-emotional, and visual domains (*p* < 0.0001), and to a lesser extent in the auditory (*p* < 0.001) and somatic domains (*p* < 0.01). Effect sizes ranged from moderate to large (r = 0.42–0.56), as detailed in Supplementary Table S2.

**Figure 2:**
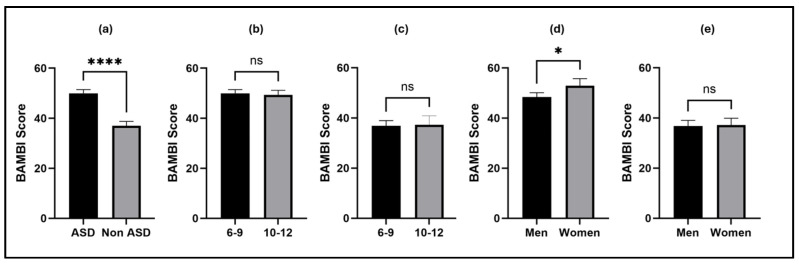
BAMBI (Brief Autism Mealtime Behaviour Inventory) scores in ASD and non-ASD populations stratified by age and sex. Results are presented for the entire sample (**a**), as well as for children with ASD (**b**,**d**) and non-ASD children (**c**,**e**). Data are presented as mean ± SEM, and comparisons were conducted using the Mann–Whitney test. * *p* < 0.05, **** *p* < 0.0001.

With respect to BAMBI scores according to diagnosis, age group, and sex of the participants (Figure 2), higher scores were observed in the ASD population compared to neurotypical children, indicating significantly greater problematic eating behaviours (*p* < 0.0001). When these results were disaggregated by age group, younger children did not show different scores compared to older children, regardless of diagnosis (*p* > 0.05). Notably, when comparing scores between male and female participants, differences were observed only within the ASD population, with higher scores among females.

When assessing the correlation between BAMBI results and sensory hypersensitivity profiles in children with ASD (Table 4), a positive, moderate, and statistically significant relationship was found between problematic behaviours and oral hypersensitivity (*p* = 0.002) and socio-emotional hypersensitivity (*p* = 0.003). A similar pattern was observed for somatic hypersensitivity, with a moderate, significant correlation (*p* = 0.025), though of lesser magnitude. On the other hand, low to very low correlations were observed for auditory, attentional, behavioural, tactile, and visual sensitivity profiles, none of which reached statistical significance. The domain showing the weakest association with reported mealtime problems was body hypersensitivity. Conversely, in the non-ASD population, only oral hypersensitivity showed a significant correlation with BAMBI scores.

## 4. Discussion

**Figure 3:**
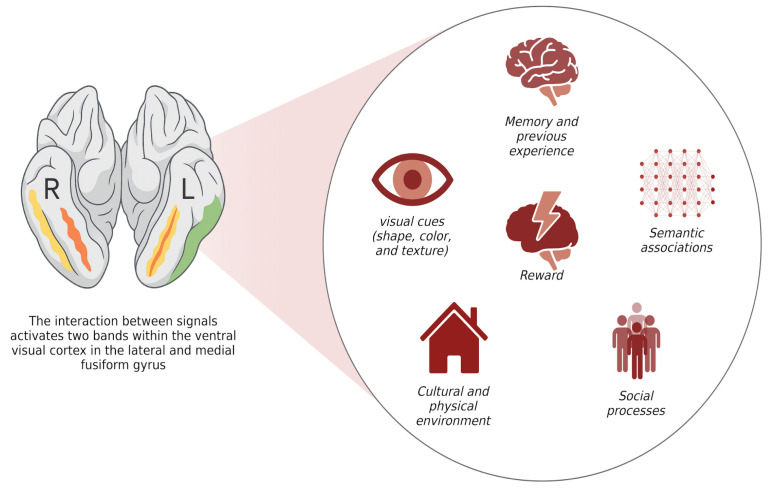
Modulatory Factors of Activation in the Ventral Visual Cortex. Among the modulatory factors are visual cues (shape, colour, texture), memory and prior experiences, the reward system, semantic associations, social processes, and the cultural and physical environment. Together, these elements contribute to establishing and refining food representations within the ventral visual cortex. As reported by Jain et al. (2023) [28], such representations are organised along gradual dimensions that reflect the prominence of food within the image (foreground vs. background), visual scale (close-up detail vs. scene context), and the presence or absence of social context. The spatial variability of these regions across individuals suggests that food representations are flexible and modulated by experience, cultural exposure, and reward history, rather than rigidly pre-specified. Figure created with BioRender^®^.

Research related to nutrition, diet, and the ASD population has become a topic of growing academic and public health interest over the past 15 years. From evaluations of nutritional interventions to analyses of public health policies aimed at enhancing the quality of life for this population, numerous studies have explored various aspects of this field [24,25]. This study confirms a high prevalence of food selectivity behaviours in children with ASD, consistent with what has been reported in the international literature [8,9,26]. We report that a substantial proportion of the diagnosed sample showed marked selectivity for food texture (78%), followed by discrimination based on colour (53%). Additionally, the practice of mealtime rituals also appeared to be associated with this phenomenon, as previously reported in Polish children with ASD [27]. These results reinforce the notion that food selectivity in children with ASD cannot be understood solely from a behavioural perspective, but instead involves specific neurocognitive mechanisms related to visual perception and multisensory integration (Figure 3). As has been recently demonstrated by independent research groups in other published neuroimaging studies, the ventral visual cortex contains specialized regions for the representation of food, with two selective bands located in the lateral and medial portions of the fusiform gyrus that respond preferentially to food stimuli over non-food stimuli, even when controlling for variables such as color, actual object size, or perspective [28].

The selectivity observed for foods such as vegetables, animal proteins, and particular dairy products may not be attributable solely to immediate sensory characteristics but also to differentiated cortical processing, the functioning of which has been shown to depend on individual and cultural experience. This reinforces the need to consider both neurocognitive and cultural dimensions in interpreting eating behaviour in ASD and to guide therapeutic interventions toward strategies that not only gradually expose children to new foods but also positively modulate reward circuits and social associations linked to eating. With respect to the first point, there are therapeutic approaches based on sensory processing that aim to address the perceptual and behavioural roots of food selectivity directly. Classical sensory integration, developed within the Ayres Sensory Integration^®^ (ASI^®^) model, has emerged as a structured, play-based intervention designed to improve sensory adaptation and regulation through controlled exposure to a range of stimuli. Another strategy used is the Sequential Oral Sensory (SOS) Approach, which follows a gradual desensitisation process through phases of visual tolerance, interaction, smell, touch, taste, and finally consumption, to increase both the variety and volume of foods accepted. Although it was not initially designed for ASD, it has been increasingly applied in this context [29]. With respect to the cultural-social dimension, qualitative evidence highlights several critical factors identified by teachers in addressing food selectivity in this context. These are mainly personal and interactive in nature, referring to the development of collaborative relationships with professionals and to the management of more effective communication of individual preferences [30]. Although the evidence has examined this dimension in depth in the pediatric population and has integrated it as a multifaceted phenomenon [31], a consensus has not yet been established on how to incorporate this element into therapeutic strategies to address food selectivity in this age group.

In addition to these social and cultural dimensions, contextual factors such as household food availability and socioeconomic status may also influence food selectivity in children with ASD. In Chile, as in many Latin American countries, dietary practices are shaped by family routines, cultural preferences, and structural inequalities that condition food access and exposure to different food types. These contextual determinants may interact with sensory and emotional factors, reinforcing restrictive patterns or limiting opportunities for dietary diversification. Considering these elements is essential for developing culturally adapted interventions and educational strategies that address both individual and environmental contributors to selective eating. Moreover, the literature documents various nutritional interventions aimed at reducing food selectivity in the ASD population, and behavioural approaches have reported significant improvements in eating behaviour. However, the increase in the number of foods consumed is not necessarily linked to healthier choices, suggesting the need for subsequent programs to improve food quality [32].

**Figure 4:**
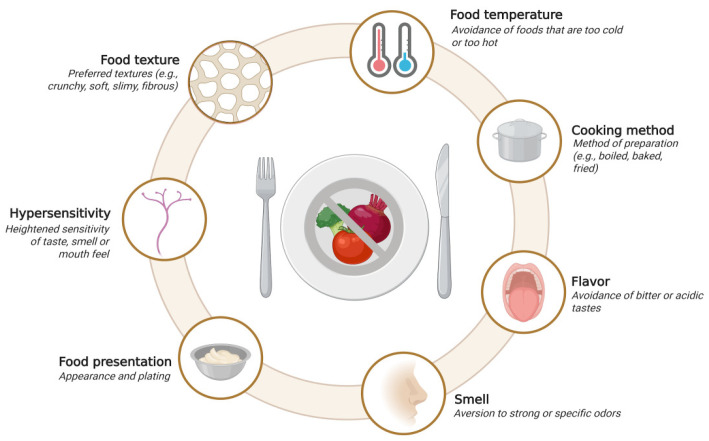
Sensory-Based Determinants of Food Avoidance and Acceptance. Sensory factors, such as food texture, temperature, cooking method, flavour, smell, presentation, and hypersensitivity, contribute to food avoidance or acceptance. These determinants interact to shape selective eating behaviours commonly observed in autism spectrum disorder. Figure created with BioRender^®^.

On the other hand, and in relation to one of the main findings of the study, we report significant correlations not only between food selectivity and the oral sensory domain but also at the socio-emotional and somatic levels, confirming previous reports in Italian and Israeli ASD populations [9,33]. These findings suggest that the response to food in this population may be modulated by hyperactivation or heightened sensitivity within cortical networks involved in the perception and processing of food-related stimuli. In children with ASD, such modulations may manifest in more rigid or atypical ways, reinforcing restricted patterns of rejection or acceptance toward certain foods or preparation characteristics, which may be exacerbated by the child’s socio-emotional or bodily state. In this context, it can be stated that sensory determinants primarily mediate food acceptance or rejection in children with ASD. As previously described, food selectivity is associated with heightened hypersensitivity to taste, smell, texture, and appearance stimuli, which may amplify the perception of aversion toward specific food characteristics (Figure 4) [34]. Attributes such as texture (e.g., crunchy, soft, fibrous), pungent odour, bitter or sour flavours, as well as temperature and method of preparation, directly influence the decision to accept or reject a food. Our results indicate that the ASD population presents greater sensory sensitivity across all dimensions, although some appear to be more closely linked to problematic eating behaviours. In this context, not only the presentation of food but also the eating environment emerges as relevant variables, interacting with perceptual and emotional processes that modulate eating behaviour. These findings reinforce the notion that interventions for food selectivity should not be limited to repeated exposure to new foods but should also include personalised strategies that account for the sensory domains involved. Moreover, innovating beyond the classical paradigm of selectivity, approaches related to emotions have already been proposed [35], creating opportunities and challenges for research on intuitive eating in this field.

Among the strengths of the present study, it should be noted that it is one of the first investigations conducted among Chilean children, a group scarcely represented in studies on the topic, thereby contributing to a broader global understanding of this phenomenon in Latin American contexts. Furthermore, not only were high rates of selectivity and associated factors reported, but the study also confirmed that socio-emotional and somatic sensitivity influence eating behaviour in children with ASD, a finding further validated when compared with a group of children without the diagnosis. In addition, none of the children were receiving nutritional counselling that could have influenced eating patterns and behaviours, regardless of diagnosis. Similarly, parents and caregivers did not have formal training in nutritional education, confirming that there was no external influence that could have modulated the reported dietary variables. In this sense, these findings may serve as a basis for future research and for assessing these items in nutritional interventions aimed at improving food selectivity. In relation to the limitations of this publication, the main one lies in the sample size. Although the calculated sample size per group was achieved, the sample does not allow these results to be extrapolated to the general population. Additionally, there was considerable heterogeneity in the baseline characteristics of the groups, ranging from sex to the type of therapy received at the foundation or educational institution they attended. However, much of this heterogeneity is inherent to studies involving ASD populations, given the spectrum within which these children develop, making it challenging not only to recruit participants with the diagnosis but even more so to assemble a homogeneous sample. Although the study was exploratory and involved a relatively small sample, potential confounding variables such as therapy type and socioeconomic context were recorded during data collection and are presented descriptively to support contextual interpretation. Owing to the exploratory scope and limited statistical power, analytical control was not feasible; this has been explicitly noted among the study’s limitations to prevent misinterpretation. Conversely, the role of occupational therapy in modulating sensory and dietary outcomes was not assessed; however, future prospective or case–control research should address this variable, given its potential influence on sensory regulation and feeding behaviour [36]. Another critical limitation concerns the reliance on parent-report instruments, such as the BAMBI and the FFQ. These tools depend on caregivers’ perceptions and recollections of the child’s eating behaviours, which may introduce recall or reporting bias. Furthermore, responses may be influenced by caregivers’ subjective interpretations of sensory-related behaviours rather than direct observation, potentially affecting the accuracy of dietary and behavioural data.

As a strategy to improve methodological designs in this area of research, it would be essential to control for the duration of therapy that children have received, particularly occupational therapy, given the results reported on sensory sensitivity and its influence on food selectivity [37,38]. In addition, it would be valuable to incorporate more and better instruments to enable a more comprehensive assessment of food selectivity. Although our study used direct reports from parents, a general instrument on consumption trends, and one focused explicitly on the ASD population, the measurement of the variable itself still presents limitations. Therefore, it would be appropriate to include additional tools and explore the phenomenon in greater depth through alternative assessment approaches [35,39]. To conclude, it is essential to encourage further research in this area within underrepresented populations and to aim for longitudinal studies that can confirm the link between socio-emotional and somatosensory hypersensitivity and food selectivity in the ASD population [40,41]. This would be both appropriate and necessary to provide evidence to support new treatment approaches aimed at improving food selectivity in this group.

## 5. Conclusions

Food selectivity in individuals with ASD is closely linked to sensory hypersensitivity, particularly in the oral, socio-emotional, and somatic domains. These findings underscore the clinical and educational relevance of incorporating sensory, emotional, and cultural factors into assessment and intervention strategies. Promoting a sensory-based, context-sensitive approach may improve dietary variety and nutritional outcomes in this population.

## Figures and Tables

**Table 1:** Sample characterization.

	*n* (%)		*n* (%)		*n* (%)
Sex		Physician		Nutritionist	
Male	33 (57.9)	Yes	5 (8.8)	Yes	0 (0.0)
Female	24 (42.1)	No	52 (91.2)	No	57 (100)
Age		Occupational Therapy		Texture	
6 to 9	42 (76.7)	Yes	21 (38.8)	Yes	32 (56.1)
10 to 12	15 (26.3)	No	36 (63.2)	No	25 (43.9)
ASD		Speech Therapist		Color	
Yes	32 (56.1)	Yes	19 (33.3)	Yes	20 (35.1)
No	25 (43.9)	No	38 (66.7)	No	37 (64.9)
School Type		Psychologist		Smell	
Traditional	48 (84.2)	Yes	20 (36.1)	Yes	25 (43.9)
Special	9 (15.8)	No	37 (64.9)	No	32 (56.1)
Teraphy		Physical Therapist		Ritual	
Yes	34 (59.6)	Yes	2 (3.50)	Yes	25 (43.9)
No	23 (40.4)	No	55 (96.5)	No	32 (56.1)

**Table 2:** Sensory Hypersensitivity Features Associated with ASD.

	ASD *n* (%)	Non ASD *n* (%)	OR	CI 95%	*p* Value
Texture					
Yes	25 (78.1)	7 (28.0)	9.18	2.78–27.5	0.0002
No	7 (21.9)	18 (72.0)	0.11	0.03–0.36	***
Color					
Yes	17 (53.1)	3 (12.0)	8.31	2.07–29.4	0.0018
No	15 (46.9)	22 (88.0)	0.12	0.03–0.48	**
Smell					
Yes	17 (53.1)	8 (32.0)	2.41	0.86–6.53	0.1783
No	15 (46.9)	17 (68.0)	0.42	0.15–1.16	ns
Ritual					
Yes	23 (71.9)	2 (8.00)	29.39	5.47–136.2	<0.0001
No	9 (28.1)	23 (92.0)	0.03	0.01–0.18	****

*p* < 0.01 **, *p* < 0.001 ***, *p* < 0.0001 ****.

**Table 3:** Dietary Intake in ASD versus Non-ASD Groups.

	ASD Mean (SD)	Non ASD Mean (SD)	*p* Value		ASD Mean (SD)	Non ASD Mean (SD)	*p* Value
Cereal grains				Legumes			
Bread	23.5 (8.4)	23.8 (7.0)	0.639	Beans	2.94 (2.9)	3.04 (2.6)	0.729
Rice	6.63 (5.6)	7.44 (4.1)	0.365	Lentils	3.53 (5.0)	3.08 (2.0)	0.802
Potatoes	8.94 (7.5)	8.72 (5.3)	0.618	Chickpeas	1.66 (3.3)	1.00 (1.8)	0.726
Pasta	10.8 (7.1)	9.76 (5.9)	0.639	Green peas	1.03 (2.3)	2.36 (3.1)	0.005
Oat	4.09 (7.5)	4.08 (7.1)	0.990				
				Lipids and Fats			
Vegetables				Butter	7.53 (9.8)	7.08 (8.6)	0.930
Lettuce	6.25 (9.5)	11.6 (7.5)	0.002	Margarine	2.63 (7.2)	6.24 (9.9)	0.048
Tomatoes	6.25 (9.3)	10.0 (8.1)	0.016	Olive oil	11.8 (12.7)	8.80 (13.1)	0.336
Carrots	9.94 (7.8)	13.4 (9.7)	0.182	Sunflower oil	13.9 (12.6)	15.7 (13.6)	0.714
Cucumber	4.75 (7.7)	6.52 (7.8)	0.132	Avocado	5.44 (7.6)	11.0 (8.3)	0.007
				Nuts	4.97 (9.3)	2.76 (4.1)	0.776
Fruits				Olives	1.97 (3.6)	1.48 (2.7)	0.928
Banana	8.28 (8.8)	10.6 (7.8)	0.130				
Apple	7.78 (8.7)	9.96 (8.7)	0.220	Sugars & Snacks			
Pear	3.00 (4.5)	2.40 (3.2)	0.931	Sacarose	7.37 (11.5)	15.4 (13.4)	0.015
Orange	7.5 (10.3)	8.72 (8.0)	0.097	Sweet Biscuits	14.8 (9.4)	18.5 (8.6)	0.121
				RTEBC	9.94 (9.1)	9.84 (9.4)	0.894
Dairy Products				Flavoured milk additives	4.78 (10.4)	7.68 (10.0)	0.089
Milk	17.5 (13.1)	18.9 (11.9)	0.868	Packaged potato chips	7.25 (7.8)	5.80 (4.4)	0.823
Yogurt	17.2 (10.3)	16.3 (8.9)	0.572	Savory crackers	7.00 (8.9)	5.76 (9.1)	0.441
Soft fresh cheese	0.94 (3.8)	1.68 (2.8)	0.037	Savoury snack sticks	2.78 (4.5)	2.52 (3.4)	0.556
Cheese	9.53 (9.8)	10.2 (7.8)	0.481				
				Beverages			
Animal Proteins				Water	23.1 (10.4)	27.4 (3.2)	0.048
Eggs	8.88 (7.8)	11.9 (8.3)	0.175	Carbonated	4.09 (7.2)	6.56 (6.2)	0.005
Chicken	8.86 (5.1)	10.4 (4.0)	0.321	Sweetened	14.5 (12.1)	14.8 (11.1)	0.721
Meat	5.50 (5.1)	9.12 (5.9)	0.021				
Pork	1.19 (1.8)	3.00 (2.5)	0.003				
Fish	3.84 (4.4)	5.48 (4.9)	0.154				

Dietary intake (mean ± SD) of selected food groups in children with autism spectrum disorder (ASD) compared to neurotypical controls (Non-ASD). Data are expressed as average monthly portions. Group comparisons were performed using the Mann–Whitney test. *p* values indicate the statistical significance of these differences. RTBEC: Ready to breakfast cereals.

**Table 4:** Correlations between Sensory Processing Domains and BAMBI scores.

Domain	r	95% CI	R^2^	*p* Value
Auditory (ASD)	0.27	(−0.09, 0.56)	0.07	0.14
Attentional (ASD)	0.17	(−0.19, 0.49)	0.03	0.36
Behavioural (ASD)	0.21	(−0.15, 0.52)	0.04	0.26
Oral (ASD)	0.53	(0.22, 0.74)	0.28	0.002
Socioemotional (ASD)	0.51	(0.20, 0.73)	0.26	0.003
Somatic (ASD)	0.40	(0.06, 0.66)	0.16	0.025
Tactile (ASD)	0.33	(−0.03, 0.61)	0.11	0.069
Visual (ASD)	0.18	(−0.19, 0.49)	0.03	0.34
Auditory (non-ASD)	0.16	(−0.25, 0.52)	0.03	0.45
Attentional (non-ASD)	0.09	(−0.31, 0.47)	0.01	0.66
Behavioural (non-ASD)	0.24	(−0.17, 0.58)	0.06	0.24
Oral (non-ASD)	0.74	(0.49, 0.88)	0.55	<0.001
Socioemotional (non-ASD)	0.29	(−0.12, 0.61)	0.08	0.16
Somatic (non-ASD)	−0.27	(−0.60, 0.15)	0.07	0.20
Tactile (non-ASD)	0.12	(−0.29, 0.49)	0.01	0.57
Visual (non-ASD)	0.09	(−0.32, 0.47)	0.01	0.68

Note. r = Pearson correlation coefficient; CI = confidence interval; R^2^ = coefficient of determination; *p* = significance level.

## Data Availability

The raw data supporting the conclusions of this article will be made available by the authors on request.

## Supplementary Materials

The following supporting information can be downloaded at: https://www.mdpi.com/article/10.3390/children12111560/s1, Table S1: Sensory_Profile_Interpretation; Table S2: Mann-U Effect.

## Abbreviations

The following abbreviations are used in this manuscript:

ASD	Autism Spectrum Disorder
ASI	Ayres Sensory Integration
BAMBI	Brief Autism Mealtime Behaviour Inventory
DSM-V	Diagnostic and Statistical Manual of Mental Disorders, Fifth Edition
FFQ	Food Frequency Questionnaire
RTEBC	Ready-To-Eat Breakfast Cereal
SOS	Sequential Oral Sensory Approach
